# Knockdown of N-Acetylglucosaminyltransferase-II Reduces Matrix Metalloproteinase 2 Activity and Suppresses Tumorigenicity in Neuroblastoma Cell Line

**DOI:** 10.3390/biology9040071

**Published:** 2020-04-04

**Authors:** M. Kristen Hall, Austin A. Whitman, Douglas A. Weidner, Ruth A. Schwalbe

**Affiliations:** 1 Department of Biochemistry and Molecular Biology Brody School of Medicine, East Carolina University Greenville, Greenville, NC 27834, USA; hallma@ecu.edu (M.K.H.); whitmana13@ecualumni.ecu.edu (A.A.W.); 2 Department of Microbiology and Immunology, Brody School of Medicine, East Carolina University Greenville, Greenville, NC 27834, USA; weidnerd@ecu.edu

**Keywords:** glycans, neuroblastoma, cell invasiveness, cell proliferation, MMP-2, EGFR, Gab2, cell adhesion

## Abstract

Neuroblastoma (NB) development and progression are accompanied by changes in N-glycans attached to proteins. Here, we investigated the role of N-acetylglucosaminyltransferase-II (GnTII, *MGAT2*) protein substrates in neuroblastoma (NB) cells. *MGAT2* was silenced in human BE(2)-C NB (HuNB) cells to generate a novel cell line, HuNB(-*MGAT2*), lacking complex type N-glycans, as in rat B35 NB cells. Changes in N-glycan types were confirmed by lectin binding assays in both cell lines, and the rescued cell line, HuNB(-/+*MGAT2*). Western blotting of cells heterologously expressing a voltage-gated K+ channel (Kv3.1b) showed that some hybrid N-glycans of Kv3.1b could be processed to complex type in HuNB(-/+*MGAT2*) cells. In comparing HuNB and HuNB(-*MGAT2*) cells, decreased complex N-glycans reduced anchorage-independent cell growth, cell proliferation, and cell invasiveness, while they enhanced cell-cell interactions. Cell proliferation, invasiveness and adhesion of the HuNB(-/+*MGAT2*) cells were more like the HuNB than HuNB(-*MGAT2*). Western blotting revealed lower protein levels of MMP-2, EGFR and Gab2 in glycosylation mutant cells relative to parental cells. Gelatin zymography demonstrated that decreased MMP-2 protein activity was related to lowered MMP-2 protein levels. Thus, our results support that decreased complex type N-glycans suppress cell proliferation and cell invasiveness in both NB cell lines via remodeling ECM.

## 1. Introduction

Neuroblastoma (NB) is a cancer derived from the sympathetic nervous system. NB is the most common extracranial solid tumor in children, resulting in approximately 15% of pediatric cancer-related deaths [1]. Recent therapeutic advances have improved the overall diagnosis of NB patients but the long-term survival of aggressive forms of NB remains poor [2]. Like other kinds of cancers, changed cell surface glycans have been linked to malignant transformation and the progression of NB [3]. However, the types of glycans involved, as well as their roles in assisting or directing cancer progression, is undefined. One group of glycans that are altered in cancer are glycans attached to proteins by *N*-acetylglucosamine-β1-*N*-asparagine (GlcNAcβ1-*N*-Asn) linkage [1,3].

There are three major types of N-glycans, referred to as oligomannose, hybrid, and complex [4]. Each type of N-glycan has a conserved pentasaccharide which is modified by the attachment of the number of branches, and length of branches. Branch points are initiated by the action of *N*-acetylglucosaminyltransferases (GnTs, encoded by *MGAT* genes) [4]. GnT-I (encoded by *MGAT1*) catalyzes the conversion of oligomannose type to hybrid type while GnT-II (encoded by *MGAT2*) catalyzes the conversion of hybrid type to complex type with two branches. Biantennary N-glycans can be processed to tri- and tetra-antennary N-glycans by the actions of GnT-IV (encoded by *MGAT4*) and GnT-V (encoded by *MGAT5*). The high activity of GnT-V has been correlated with a metastatic phenotype [5], and led to the use of swainsonine in clinical trials [6,7,8]. Swainsonine prevents the conversion of hybrid to complex types of N-glycans and thereby raises the levels of hybrid-type N-glycans [9]. The initial clinical trials [6,7] appeared quite promising but the later trials [10,11] proved the drug to be toxic, halting clinical trials. Increased action of GnT-V was also observed with a more aggressive NB phenotype, since increased levels of β1,6-branched N-glycans, along with a decline in hybrid-type N-glycans, were found in cells derived from high-risk neuroblastoma (NLF) compared to cells derived from low-risk neuroblastoma (SY5Y) [12]. Further, a rat B35 NB cell line was more aggressive when complex type N-glycans were present, while raised levels of hybrid decreased aberrant cellular properties [13].

Receptor tyrosine kinases (RTKs) are cell surface receptors that receive and transduce signals which are vital in survival, growth, and differentiation of many normal and malignant cells. Anaplastic Lymphoma Kinase (ALK) was upregulated in advanced/metastatic neuroblastomas [14,15], and furthermore, treatment of ALK-positive neuroblastoma cells with tunicamycin, an inhibitor of N-glycosylation, impaired the pro-survival signaling pathway [16]. Another RTK that was found to be present and at high levels in NB cell lines and primary tumors was the epidermal growth factor receptor (EGFR) [17,18]. Others have also reported that high-risk neuroblastoma associated with MYCN amplification cooperates with Gab2 [19], a scaffold protein that amplifies EGFR signaling [20,21]. Matrix metalloproteases (MMPs) have been connected to cell proliferation and invasiveness as they degrade proteins in the extracellular matrix and increase the availability of growth factors and cytokines to RTKs [22,23].

Previous studies from our lab showed that cellular behavior was impacted in engineered Chinese Hamster Ovary (CHO) [24], and rat NB [25] cell lines by silencing the *MGAT2* gene, which resulted in glycosylation mutant cell lines that were unable to convert hybrid to complex types of N-glycans. Further, it was found that the presence of complex type N-glycans enhanced NB tumor cell properties [13]. Since neuroblastomas are biologically heterogeneous, we aimed to determine whether increased hybrid and/or oligomannose types of N-glycans and decreased complex type N-glycan via the GnT-II knockdown, suppressed cell proliferation, cell association and dissociation, and cell invasiveness in a human NB cell line, similarly as in rat NB cells. Also, to identify whether MMP-2, EGFR, and Gab2 protein levels were lowered in the two distinct NB cell lines when complex type N-glycans were decreased. Thus, this study addressed whether modifications in the N-glycosylation pathway may provide a biologically based therapeutic agent for NB.

## 2. Materials and Methods

### 2.1. Cell Lines

Human BE(2)-C (HuNB) and rat B35 (NB_1) neuroblastoma (NB) cells were purchased from American Type Culture Collection (Manassas, VA, USA) and used to engineer the human, HuNB(-*MGAT2*), and rat, NB_1(-*MGAT2*) glycosylation mutant cell lines, and also, NB cell lines stably expressing wild-type (WT) or N220/9Q (DM) Kv3.1b proteins [13,25]. Cultured NB cell lines were maintained in DMEM, including 10% FBS, 50 U/mL penicillin, and 50 µg/mL streptomycin at 37° in a 5% CO_2_ atmosphere. In short, CRISPR-Cas9 technology was used to silence *MGAT2* in the HuNB cell line to produce HuNB(-*MGAT2*), as we previously described for creating the rat NB_1(-*MGAT2*) cell line [25]. Of note, the sgRNA oligonucleotides (5′-CACCGTTCCGCATCTACAAACGGA-3′ and 5′-AAACTCCGTTTGTAGATGCGGAAC-3′) were identical to those used to silence *MGAT2* in Chinese hamster [24] and rat [25] cells. Sequencing of the genomic DNA fragment verified that the gene was silenced from nine separate cell clones. The HuNB(*-MGAT2*) cell line was rescued by transient transfection with pCMVSport6 recombinant vector coding the mouse MGAT2 cDNA (Thermofisher Scientific, MA, USA) using the Lipofectamine^®^ 2000 (Thermofisher Scientific, MA, USA) protocol [13]. The mouse *MGAT2*-pCMVSport6 vector [26] was kindly provided by Dr. Pamela Stanly, College of Albert Einstein. 

### 2.2. Whole Cell Lysates and Total Membranes

Various cell lines were harvested and washed with PBS on the cell dish. Washed cells were resuspended in RIPA buffer (PBS, 1% Triton X-100, 0.5% sodium deoxycholate, 0.1% SDS) plus protease inhibitor cocktail set III (EMD Biosciences, San Diega, CA, USA), and then cells were placed into a microcentrifuge tube, as described [27]. A 1 mL syringe with a 21 gauge needle was used to shear the DNA. Following incubation of samples on ice for 30–60 min, they were centrifuged at 15,000 × g for 20 min at 4 °C, and the supernatant was collected. In brief, total membrane isolations were as we previously described [28]. Cells were homogenized in lysis buffer (10 mM Tris, pH 7.4; 250 mM sucrose, 5 mM EDTA; protease inhibitor cocktail set III 1:500) (Calbiochem). Homogenized cells were spun at low speed for 10 min, and then collected supernatants were centrifuged at high speed for 1 h at 4 °C. The pellet was then resuspended in lysis buffer. In both cases, protein levels were determined by a modified Lowry assay. Aliquots of each sample were denatured and reduced in SDS-PAGE sample buffer for lectin and western blotting while the remainder was stored at −20 or −80 °C until needed.

### 2.3. Lectin Binding Analysis by Flow Cytometry

A fluorescein-tagged lectin (10 µg/mL, Vector Laboratories, Inc., Burlingame, CA, USA), Phaseolus vulgaris leucoagglutinin (L-PHA), Galanthus nivalis lectin (GNL), Phaseolus vulgaris erythroagglutinin (E-PHA), or concanavalin A (ConA) was incubated with cells for 15 min at room temperature. A FACS Vantage flow cytometer (Becton Dickinson Biosciences, San Jose, CA, USA) was used with 488 nm laser excitation and emission centered at 530 nm to acquire fluorescence intensity of tagged cells. Mean fluorescent values were determined from histogram plots of fluorescence emission.

### 2.4. Glycosidase Digestions of Membrane Proteins

Total NB membranes were treated with glycosidases as described [29]. Total membranes (5 g/L) were incubated with 50 U/μL Endo H, 20 U/μL PNGase F, and 0.83 U/μL neuraminidase in accompanied buffers (New England Biolabs, Ipswich, MA, USA). Reactions were performed overnight at 37 °C and halted by the addition of reducing SDS-PAGE sample buffer.

### 2.5. Western and Lectin Blots

Total cell membrane and whole cell lysate samples were evaluated by western and lectin blotting [25,29]. Samples of HuNB(*-MGAT2*) cells transiently expressing MGAT2 were harvested at about 72 h for Western and lectin blots. Proteins were separated on 10% SDS gels for 1.7 h at 20 mA, and then blotted to PVDF membranes (Millipore, Billercia, MA, USA) for 2.5 h at 250 mAmps. Blotted membranes were incubated with primary and secondary antibodies and then visualized using NBT/BCIP. The primary antibodies include: mouse anti-Kv3.1 b antibody (Neuromab, Davis, CA, USA), rabbit anti-EGFR (Cell Signaling Tech, Danvers, MA, USA), rabbit anti-Gab2 (Cell Signaling Tech, Danvers, MA), and mouse anti-MMP-2 (Novus Biologicals, Centennial, CO, USA). Biotin-conjugated L-PHA, E-PHA, or GNL (Vector Laboratories, Burlingame, CA, USA) lectins were employed to probe lectin blots.

### 2.6. Anchorage-independent Growth

Soft agar assay was employed to measure anchorage-independent growth [30]. In short, 1% low melting temperature agarose in DMEM supplemented with 10% FBS was aliquoted into a 6-well plate. After solidification, equal parts cell suspension was mixed with 1% low melting noble agar and added to the top of the solidified base layer. Cell plates were cultured for 14 days. An Olympus IX73 microscope with a 4 × objective was used to acquire images. Area of the cell colonies and the number of cell colonies were determined using ImageJ software.

### 2.7. Cell Proliferation Assay

The 5-bromo-29 deoxyuridine (BrdU) proliferation assay (Millipore, Billerica, MA) was used to measure cell proliferation using the accompanied manufacturer’s protocol, as we previously described [13]. HuNB(*-MGAT2*) cells transiently transfected for 72 h were used for the BrdU assay. Shortly, BrdU was added to plated cells in a 96-well plate. After 24 h, cells were fixed for 30 min at room temperature, incubated with anti-BrdU monoclonal antibody for 1 h at room temperature, followed by secondary goat-anti mouse IgG peroxidase conjugate for 30 m. After incubation of cells with peroxidase substrate for 30 min, absorbance at 450 nm was measured using a Multiskan FC plate reader (Fisher Scientific, Atlanta, GA, USA).

### 2.8. Cell Dissociation Assay

Cells were grown to confluency on 35 mm CellBind culture dishes (Corning, Corning, NY, USA), as described [13]. HuNB(*-MGAT2*) cells transiently transfected for 24 h were used for the cell dissociation assay. Cells were resuspended in serum-free media following two rinses with media, and then cells were detached by one complete rotation with a cell scraper. Using a 1 mL pipet tip, detached cells were dissociated by pipetting 14 times. An Olympus IX 71 microscope using a 20 × objective was used to acquire images (25–30 fields/dish). Image J software was employed to measure the area of cell aggregates (≥10 cells/aggregate).

### 2.9. Hanging Drop Aggregation Assay

Cell association was examined as previously described [28]. In brief, NB cells were collected by detaching cells with trypsin, and then washed twice in PBS and resuspended in DMEM (Mediatech) at a concentration of 5.0 × 10^5^ cells/mL. A drop (30 µL) of cell suspension was placed on the inner surface of the lid of a 24-well cell culture dish. The lid was placed over the wells, containing 0.5 mL of PBS. The cells were cultured for 96 h in the hanging drop at 37°. The weak cell aggregates were disrupted by pipetting up and down 10 times using a standard 200 µL pipet tip. Images (3 fields/well) were captured with an Olympus IX50 microscope (Olympus, Center Valley, PA, USA) using a 10 × objective. Image J software was used to determine the area of particles (>10 cells per aggregate or greater than 2000 pixels).

### 2.10. Cell Wound Healing Assays

Media was removed from a confluent dish of cells and wounds were made in the cell monolayer using a beveled 200 µL pipet tip as described [31]. Cells were rinsed two times with media, and subsequently, an Olympus IX 71 microscope using a 4× objective was used to capture images at 0 and 19 h. The percent of wound closure (arbitrary units, AU) was ascertained by determining the difference between the initial and final widths of the cell wound and then multiplying by 100%.

### 2.11. Matrigel Invasion Chamber Assay

Cell invasion was assayed using the BD Falcon matrigel invasion chambers (BD Biosciences, San Jose, CA, USA) and the accompanied manufacturer’s instructions were followed. HuNB(-MGAT2) cells transiently transfected for 72 h were used for the cell invasion assay. Briefly, the matrigel of 24-well plates were rehydrated by addition of DMEM for 2 h at 37°. Cells (5.0 × 10^4^) in serum-free DMEM (500 µL) were plated in each transwell insert. NIH-3T3 conditioned media (500 µl) was added to the lower chamber of the plate and cultured at 37 °C for 24 h. Cells in the interior of the transwell insert were gently removed, and cells on the bottom surface of the insert were fixed with 100% methanol and stained with 1% Toluidine blue. Three experiments were conducted in quadruplicate. Cells from five fields per membrane insert were counted using a Nikon TMS microscope (Nikon, Chiyoda, Japan). Each of the cell lines were normalized to the HuNB cell line. Images were captured using an Olympus IX71 microscope (Olympus, Center Valley, PA, USA).

### 2.12. Three-dimensional (3D) Spheroid Assay

A 3D Spheroid assay was also used to measure cell invasiveness as we previously described [13]. Briefly, spheroids were formed using the hanging drop method which involved seeding cells (5 × 10^5^ c/mL) in 30 µL drops onto the lid of a 100 mm culture dish, and then allowed to incubate for 96 h at 37°. Spheroids were collected by placing a drop in a microcentrifuge tube and allowing spheroids to settle for 10 min. The drops were then combined with 100 µL of Matrigel (Fisher Scientific, Atlanta, GA, USA). The Spheroid/Matrigel mixture (40 µL) was pipetted into the center of the wells of a 24-well plate (4 wells per cell line) via pre-chilled pipet tips. Matrigel was allowed to polymerize by incubation at 37° for 30 min. Then, media (1 mL) was added to culture and incubated for at 37° for 72 h. A 10 × objective on an Olympus IX73 microscope was used to capture images. ImageJ software was utilized to measure the sphere and invasive areas. Dividing invasive area by sphere area reports the cell invasiveness.

### 2.13. Gelatin Zymography

Cells were plated near confluency in serum-free media and incubated for 48 h. After the incubation period, the serum-free media was collected and concentrated, referred to as conditioned serum-free media. Protein levels were determined using the Lowry assay. Non-reducing buffer (2 ×) was added to concentrated conditioned media. Samples were electrophoresed using 10% SDS acrylamide gels containing 4 mg/mL gelatin at 20 mA for 1.5 h. Gel containing separated proteins were washed twice with washing buffer (2.5% Triton X-100, 50 mM Tris-HCL, pH 7.5, 5 mM CaCl_2_, 1 µM ZnCl_2_) for 30 min, and then rinsed in incubation buffer (1% Triton X-100, 50 mM Tris-HCL, pH 7.5, 5 mM CaCl_2_, 1 µM ZnCl_2_) for 5–10 min with gentle agitation at 37 °C. The gel was then incubated in fresh buffer at 37 °C with slight agitation. After 24 h, the gel was stained with Coomassie Blue for 30–60 m on a rotator. Bands were then visualized by incubation of gel in a de-staining solution.

### 2.14. Data Analysis

Image J software (developed by National Institute of Health and Laboratory for Optical and Computational Instrumentation, USA) was employed for data analysis, including measurements of areas of cell clusters, colonies, spheroids, and spheroid invasion. Adobe Photoshop (Adobe Inc., San Jose, California, USA) was employed to measure cell wounds. Origin 7.5 (OriginLab, Northampton, MA, USA) was used for graphics and statistics. UN-SCAN-IT software (Silk Scientific, Inc., Orem, Utah, USA) was employed to measure immunoband intensities to determine MMP-2 protein levels, and also, to measure MMP-2 activity (e.g., band intensity was increased as gelatin levels were reduced). Data is presented as the mean SE, where n denotes the number of observations. The unpaired Student’s *t*-test was used for comparing mean values, unless indicated.

## 3. Results

### 3.1. Establishment of NB Cell Models Lacking GnT-II Expression

**Figure 1:**
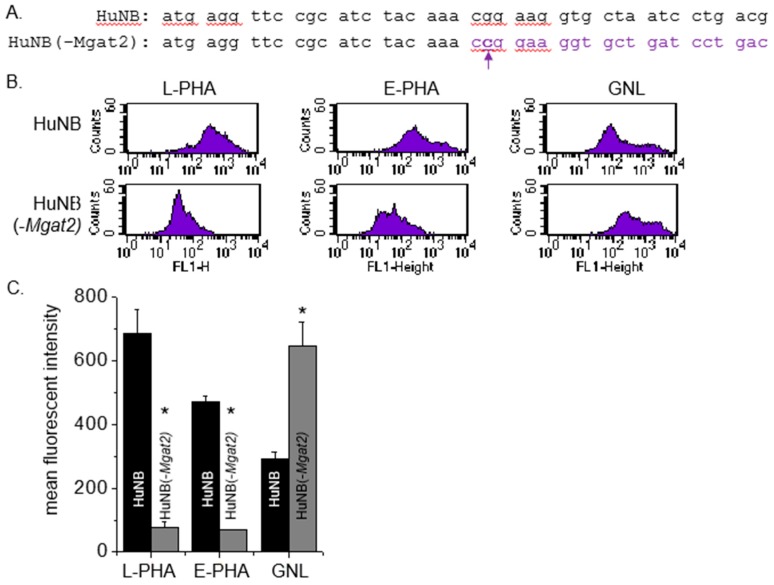
Construction and characterization of an NB cell line with an introduced frameshift in *MGAT2*. The *MGAT2* gene was silenced in the HuNB cell line using CRISPR/Cas9 technology. Coding sequences of the *MGAT2* gene from 1 to 42 is shown for the parental and N-glycosylation mutant cell lines *(***A**). The mutant cell line has a c inserted following the 22nd nucleotide, which is denoted in bold purple font. The purple font reveals the different codons in the sequence caused by the frameshift mutation. Typical flow cytometry plots of fluorescently labelled L-PHA (left panels), E-PHA (left middle panels), and GNL (right middle panels) bound to HuNB (top panels) and HuNB(-*MGAT2)* (bottom panels) cell lines are shown (**B**). Mean fluorescence intensity values of all three lectins bound to each of the cell lines were obtained from 4 experiments (**C**). * *p* < 0.01.

The CRISPR/Cas9 technique [32] was employed to engineer a human NB (HuNB) cell line with *MGAT2* (encodes GnT-II) silenced, referred to as HuNB(-*MGAT2*). Previously, we used this technique to construct a rat NB (NB_1) cell line lacking GnT-II, called NB_1(-*MGAT2*) [25]. Since DNA sequences are conserved in this region between human and rat, the gRNA pair employed was identical. A HuNB(MGAT2) cell clone was expanded, and then the genomic DNA was isolated, amplified, and sequenced. Nine recombinant vectors containing the DNA fragment revealed that the *MGAT2* gene had a C residue inserted after the 22nd nucleotide residue (Figure 1A). This frameshift mutation was identical to that in the NB_1 (-*MGAT2*) cell line [25]. An alternative in the frame start site is not present until amino acid residue 275.

### 3.2. Lectin Binding Assays of Cell Lines

**Figure 2:**
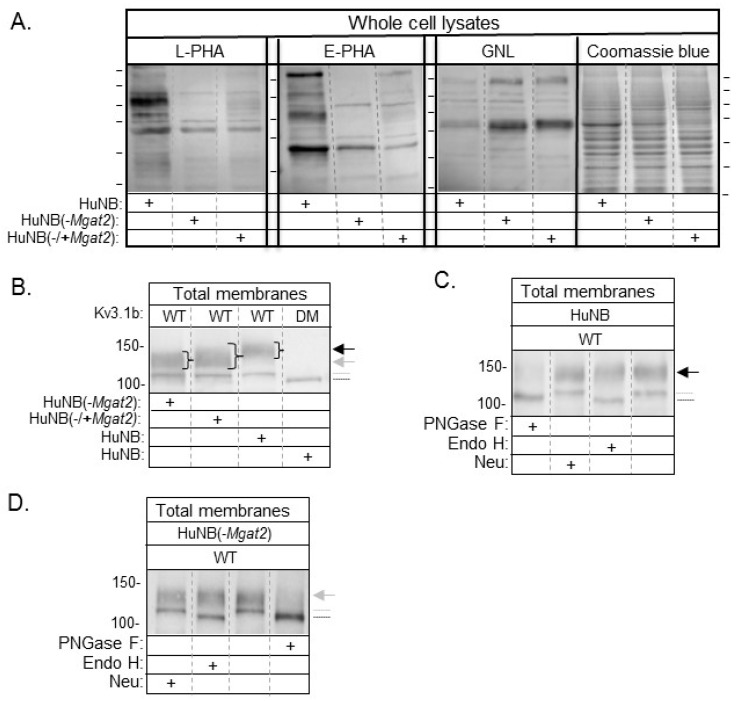
Identification of reduced levels of complex type N-glycans in NB cells with *MGAT2* silenced. Lectin blots of whole cell lysates from HuNB and HuNB(-*MGAT2)* cell lines, along with the later cell line transiently transfected with *MGAT2* to create the rescued cell line, called NB_1(-/+*MGAT2*) (**A**). Proteins separated on membranes were probed with L-PHA, E-PHA, and GNL, as indicated. Coomassie blue-stained SDS gels show that similar levels of protein were loaded per lane. Horizontal lines adjacent to blots and gel indicate molecular weight standards in kDa: 250, 150, 100, 75, 50, and 37, from top to bottom. Western blot of total membranes from HuNB, HuNB(-*MGAT2*), and HuNB(-/+*MGAT2*) cell lines transfected with glycosylated Kv3.1b (WT), along with HuNB transfected with unglycosylated Kv3.1b (DM) (**B**). (+) denotes the cell line studied. Black and gray arrows reflect complex and hybrid types of N-glycans respectively, attached to the Kv3.1b protein. The gray dotted line denotes oligomannose type glycans attached to Kv3.1b, and the black line denotes unglycosylated Kv3.1b. Western blots of WT Kv3.1b from total membranes of HuNB (**C**), and HuNB(-*MGAT2*) (**D**) cells treated with glycosidases, as indicated. Numerical values adjacent to western blots denote molecular weight markers.

Lectin binding assays were utilized to evaluate the types of N-glycans expressed on the cell surface. The glycan binding preference of *Phaseolus vulgaris* Leucoagglutinin (L-PHA), *Phaseolus vulgaris* Erythoagglutinin (E-PHA), and *Galanthus nivalis* Lectin (GNL) is more highly towards complex type N-glycans, bisecting N-acetylglucosamine N-glycans, and α-mannose residues, respectively [33]. Representative flow cytometry histograms show that more L-PHA and E-PHA lectins bind to the cell surface of HuNB cells than the HuNB(-*MGAT2*) cells (Figure 1B). In contrast, there was a reduction in the GNL bound to the plasma membrane of the HuNB cell line compared to the HuNB(-*MGAT2*) cell line. The bar graph showed that binding of L-PHA and E-PHA were at least five-fold higher for the parental cell line than the glycosylation mutant, while the binding of GNL was reduced by half (Figure 1C).The difference in GNL binding between HuNB cells and HuNB(-*MGAT2*) cells was like that observed in the parental and glycosylation mutant lacking *MGAT2* in rat NB [13,25] and CHO [24] cell lines. Lectin blotting verified the flow cytometry observations, such that band intensities detected by L-PHA and E-PHA were much higher in the HuNB cell line than the HuNB(-*MGAT2*) cell line, while they were of lighter intensity in the HuNB cell line using GNL (Figure 2A). Additionally, all three lectin blots showed that transient transfection the HuNB(-*MGAT2*) cell line with mouse *MGAT2*, denoted as HuNB(-/+*MGAT2*), had binding affinities in between the parental and glycosylation mutant cell lines. The coomassie blue-stained gels indicate that similar levels of protein were loaded for all three cell lines.

### 3.3. Western Blotting of Cell Lines

Previously, we demonstrated that WT Kv3.1b, a voltage-gated K+ channel, is substrate for GnT-II in rat brain [34], and various mammalian cell lines [25,28,35,36]. Further mutation of Asn220 and Asn229 to Gln residues produced unglycosylated Kv3.1b protein, called DM [37]. Western blots of total membranes from HuNB and HuNB(-*MGAT2*) cells stably expressing WT Kv3.1b produced two immunobands, while those expressing DM generated one immunoband (Figure 2B). The electrophoretic mobility of the upper band for WT Kv3.1b protein was slower when expressed in HuNB than that in HuNB(-*MGAT2*). Further, when *MGAT2* was transiently expressed in the HuNB(-*MGAT2*) cell line, the upper immunoband became more diffused with its center running slower than that in HuNB(-*MGAT2*) cells. In all cases, the lower immunobands of WT Kv3.1b protein migrated the same distance, but slightly slower than the unglycosyated Kv3.1b (DM) protein. Previously, it was shown to consist of Kv3.1b with oligomannose N-glycans [35]. Of note, the level of Kv3.1b with oligomannose N-glycans appeared to be increased in the mutant cell line relative to the parental cell line. To further verify that the N-glycans attached to Kv3.1b were of complex and hybrid types of N-glycans in HuNB (Figure 2C) and HuNB(-*MGAT2*) (Figure 2D) cells respectively, total membranes were treated with various glycosidases, and then analyzed by western blotting. In both cases, treatment of total membranes with PNGase F (cleaves all types of N-glycans) produced a single immunoband which migrated like the DM Kv3.1b protein, and furthermore, treatment of both membranes with endoglycosidase H (Endo H, cleaves oligomannose type N-glycans) revealed that the upper band was resistant to cleavage, but the lower band was sensitive. Since the upper band was resistant to Endo H, it indicates that the hybrid N-glycan was processed by the medial Golgi complex enzymes of the N-glycosylation pathway [38]. A slight shift in the upper immunoband was observed in HuNB cells when membranes were treated with neuraminidase (Neu, removes sialic acid residues at the end of chains) but virtually undetected in HuNB(-*MGAT2*) cell membranes. Taken together, the results strongly support that *MGAT2* was silenced in the HuNB cell line, as we previously showed in an engineered rat NB cell line [13,25], and furthermore, the hybrid type N-glycan has less than four mannose residues for the Kv3.1b protein. Of note, decreased complex type N-glycans correlated with predominantly increased hybrid type N-glycans, and some oligomannose type N-glycans. As such, both sets of parental, HuNB and NB_1, and glycosylation mutant, HuNB(-*MGAT2*) and NB_1(-*MGAT2*), cell lines will be employed to evaluate the role of GnT-II (*MGAT2*) protein substrate in altering NB cellular properties.

### 3.4. Decreased Complex Type N-glycans Reduce Cell Growth and Proliferation

**Figure 3:**
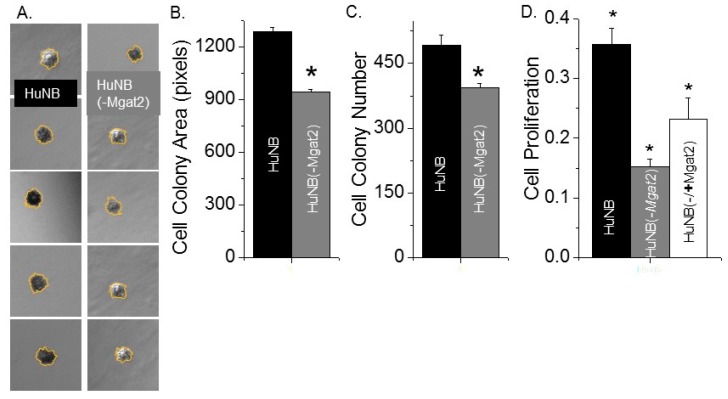
Increased levels of hybrid and oligmannose types of N-glycans reduced HuNB cell growth. Typical DIC micrographs of cell colonies of HuNB (left panels) and HuNB(-*MGAT2*) (right panels) cell lines from the anchorage-independent cell growth assay (**A**). Bar graphs show the mean cell colony areas (**B**), and cell colony number per experiment (**C**) of each cell line from 3 experiments. * *p* < 0.02. The cell-attached proliferation assay was conducted three times in triplicate for the various NB cell lines (**D**). Mean differences were compared using one-way analysis of variance (ANOVA) followed by Bonferroni’s post-hoc tests. A value of *p* < 0.05 was considered significant (*).

An anchorage-independent cell growth assay was employed to ascertain the impact of complex type N-glycans on the ability of HuNB cells to grow without attachment and spreading onto a substratum. After 14 days of cell growth, images were captured from three wells for each of the cell lines (Figure 3A). During this time course, the HuNB cell line formed significantly larger (Figure 3B) and more (Figure 3C) cell colonies than the HuNB(-*MGAT2*) cell line. These results indicated that increased hybrid and oligomannose types of N-glycans suppress independent anchorage cell growth.

Cell-attached proliferation was evaluated by measuring the level of BrdU incorporated into the DNA during its replication process on cell culture plates at about 75% confluency. DNA replication occurred at a faster rate for HuNB cells than for HuNB(-*MGAT2*) (Figure 3D). Further, when the HuNB(-*MGAT2*) cell line was transiently transfected with *MGAT2* cDNA, the incorporation of BrdU into genomic DNA was increased. These results showed that the elimination of complex-type N-glycans in HuNB cells markedly reduced their ability to proliferate by at least 42% of the parental cell line. Further, ectopic expression of *MGAT2* in the glycosylation mutant cells without complex type of N-glycans enhanced cell proliferation to at least 65% of the parental cell line, and thereby strongly supported that the decline in HuNB cell proliferation is due to enhanced expression of hybrid and oligomannose types of N-glycans.

### 3.5. Decreased Complex-Type N-glycans Influence Cell–Cell Adhesion

**Figure 4:**
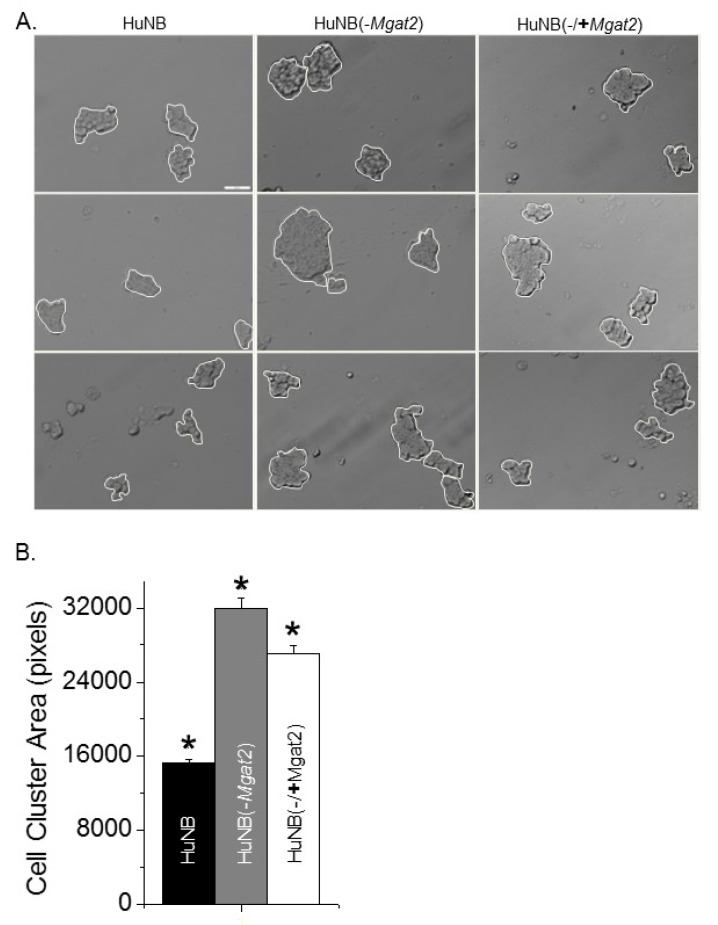
Higher levels of hybrid and oligomannose types of N-glycans in NB cells decrease disruption of cell–cell adhesion. Characteristic DIC images obtained from HuNB (left panels), HuNB(-*MGAT2*) (middle panels), and HuNB(-/+*MGAT2*) (right panels) cell lines *(***A***)*. Scale bar is 50 µm. Cell clusters of greater than 5 cells, as encircled in white, were analyzed. The mean area of cell clusters from three cell dissociation experiments for each cell line were analyzed (**B**). * *p* < 0.01. Mean differences were compared using One-way ANOVA followed by Bonferroni’s post-hoc tests was used to compare differences in mean values. A value of *p* < 0.01 was considered significant (*).

**Figure 5:**
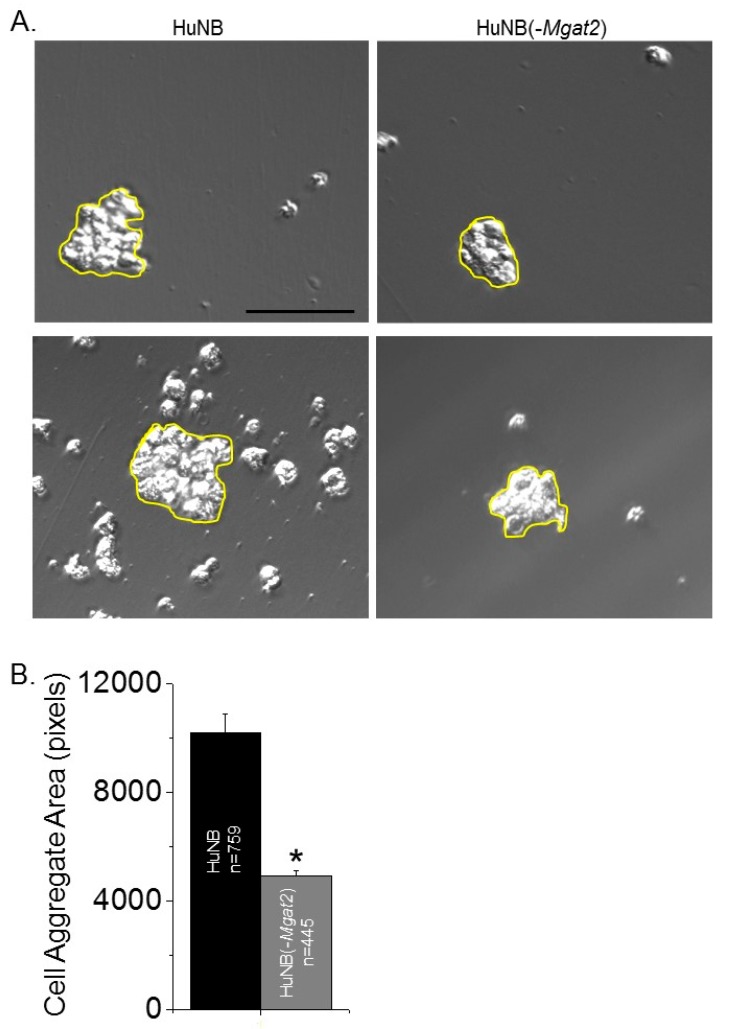
Decreased complex type N-glycans in NB cells decreases cell–cell association. Selected micrographs acquired from HuNB (left panels), and HuNB(-*MGAT2*) cell lines from the hanging drop assay *(***A***)*. Scale bar is 100 µm. Cell areas at or greater than 2000 pixels, encircled in yellow were analyzed. The mean area of cell clusters from three experiments were determined for each cell line (**B**). The unpaired Student’s t-test was used for comparing mean values at *p* < 0.00001 (*).

Cell adhesion was evaluated by measuring the dissociation and formation of cell aggregates. Cell monolayers were detached and dissociated by pipetting up and down 10 times from fully confluent cell culture dishes. Representative images show that the size of the cell clusters from the HuNB cell line were smaller than the HuNB(-*MGAT2*) cell line, and when the latter cell line was transiently transfected with MGAT2, the cell clusters were reduced (Figure 4A). The cluster size was increased about two-fold when Gn-TII activity was abolished relative to the parental cell line, while the addition of Gn-TII activity to the NB(-*MGAT2*) cell line reduced the size by at least 15% (Figure 4B). Cell–cell association was evaluated using the hanging drop assay. The typical size of cell clusters formed after 24 h in the hanging drop are shown (Figure 5A). Encircled cell aggregates of greater than 2000 pixels were used for analysis. The mean particle size of cell aggregates was reduced by at least 50% when Gn-TII activity was eliminated (Figure 5B). Thus, hybrid and oligomannose types of N-glycans reduced dissociation and formation of cell clusters of NB cells.

### 3.6. Cell Invasiveness Was Weakened by Increased Levels of Hybrid and Oligomannose Types of N-glycans

**Figure 6:**
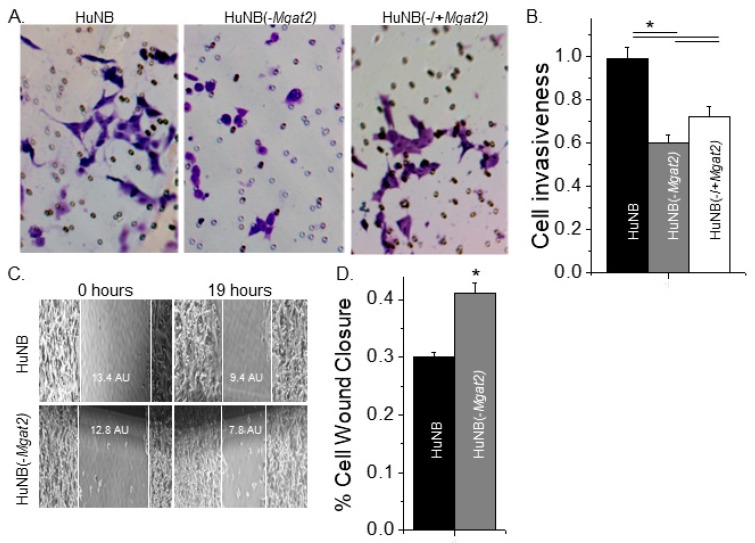
Higher levels of hybrid and oligomannose types of N-glycans diminished cell invasiveness. Selected images obtained from matrigel invasion assays of HuNB (left panels), HuNB(-MGAT2) (middle panels), and HuNB(-/+MGAT2) (right panels) (**A**). Bright purple invasive cells and pores in the membrane are visible. The mean number of invasive cells per well from the indicated cell lines were normalized to that of the HuNB cell line (**B**). Cell invasiveness was determined from three experiments performed in quadruplicate for each cell line. Micrographs of cell wound healing assays were acquired for the HuNB (upper panel) and the HuNB(-MGAT2) (lower panel) at 0 and 19 h (**C**). White lines at the edge of the monolayer are used to denote the width of the cell wound. AU represents the arbitrary unit. The bar graph indicates the percent of cell wound closure in 19 h from 11 experiments with at least 5 wounds per experiment of each cell line (**D**). Mean values were compared using the unpaired Student’s *t*-test at *p* < 0.05 (*).

Matrigel cell chambers were employed to evaluate cell invasiveness of the NB cell lines. Conditioned medium from NIH3T3 cell cultures was used to attract cells to enter the bottom of the chamber. After 24 h, membrane inserts were collected, washed, and mounted on microscope slides, and then images of the membranes were acquired. It was observed that more HuNB cells migrated through the matrigel than HuNB(-*MGAT2*) cells, and that expression of GnT-II in the latter cell line (HuNB(-/+*MGAT2*)) increased cell invasiveness, as viewed in typical micrographs (Figure 6A). The mean number of invasive cells were determined for each cell line, and then normalized to the HuNB cell line, showing that HuNB(-*MGAT2*) cells were much less invasive (Figure 6B). However, the HuNB(-*MGAT2*) cells became more invasive when they were transfected with *MGAT2*. 

To discriminate between cell migratory rates and invasiveness, cell wound healing assays were conducted. Once cell confluency was reached, scratches were introduced into monolayers, and images of cell wounds were captured at 0 and 19 h under standard cell culture conditions (Figure 6C). These micrographs show one scratch obtained for each cell line from four independent experiments. The percent of cell wound closure was slightly higher for HuNB(-MGAT2) cells than HuNB cells (Figure 6D). Although cell invasiveness was reduced by about 59% when complex type N-glycans were decreased, cell migratory rates were only slightly increased.

### 3.7. Decreased Complex N-glycans Reduce Tumor Spheroid Formation

**Figure 7:**
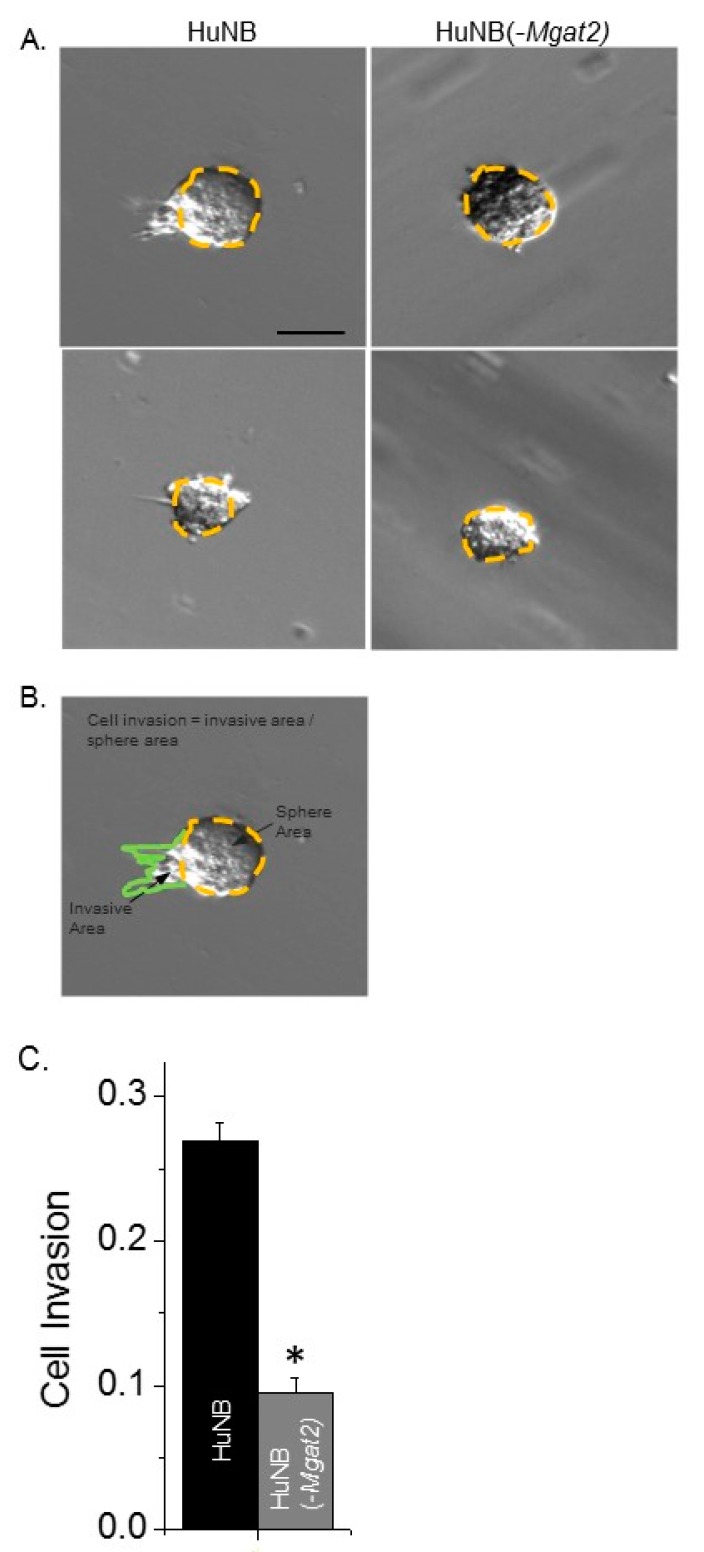
Decreased complex type N-glycans lessens cell invasiveness of three-dimensional (3D) spheroids derived from HuNB cells. After 13 days, images of invading spheroids were acquired from HuNB and HuNB(-*MGAT2*) cell lines. Representative micrographs are shown of HuNB (left panel) and HuNB(-*MGAT2*) (right panel) (**A**). Scale bar is 100 µm. Cell invasion was determined by dividing invasive area (green line) by the sphere area (yellow dotted line) (**B**). The mean cell invasion area was calculated from three experiments of at least 172 spheroids (**C**). * *p* < 0.00001.

Cell invasiveness was also evaluated using the three-dimensional tumor spheroid invasion assay. Spheroids were added in cell dishes containing Matrigel and cultured for 13 days. Images of the spheroids were then acquired for the HuNB and HuNB(-*MGAT2*) cell lines (Figure 7A). Measured sphere and invasive areas were divided to directly compare cell invasiveness of at least 172 spheroids from three experiments (Figure 7B). The cell invasiveness of the HuNB cell line was about 2.8-fold greater than the HuNB(-*MGAT2*) cell line (Figure 7C). Taken together, the two independent invasive assays strongly support that complex-type N-glycans contribute to the cell invasive phenotype of NB.

### 3.8. Increased Hybrid and Oligomannose Types of N-glycans Lower MMP-2 Protein Activity and Levels

**Figure 8:**
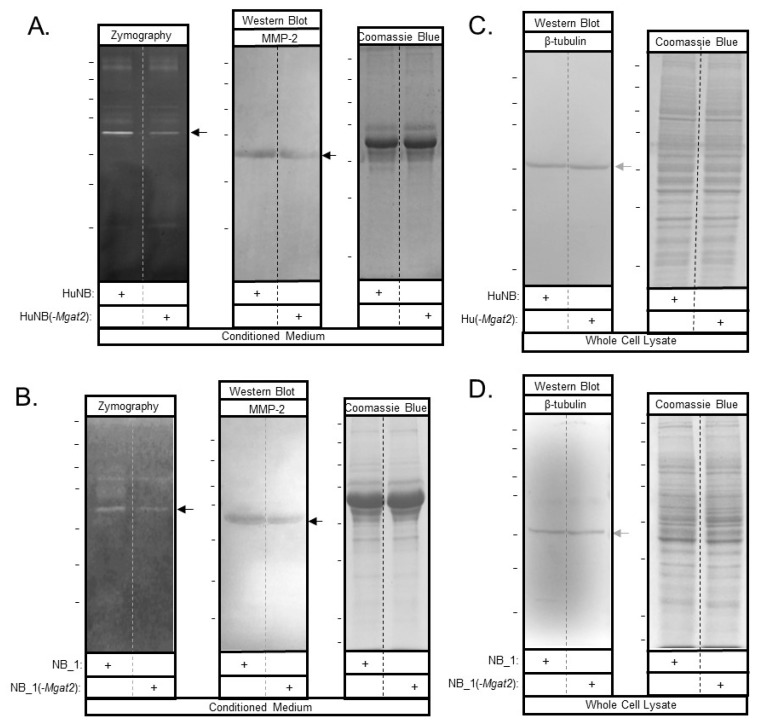
MMP-2 protein levels are lowered with increased hybrid and oligomannose types of N-glycans in NB cell lines. Conditioned medium was harvested from confluent plates. MMP-2 was detected by zymography (right panels), and western blots (middle panels) of the human and rat NB cell lines (**A**,**B**). Coomassie blue-stained gel (left panels) indicates that similar amounts of protein were present in the conditioned medium (**A**,**B**). Western blot of β-tubulin (right panels) and coomassie blue-stained gel (left panels) of whole cell lysates indicate that similar amounts of cells were used to generate MMP-2 in conditioned medium. (**C**,**D**). The reduction in activity and immunoband intensities between HuNB and HuNB(-MGAT2) were 29% ± 4% (n = 7) and 37% ± 11% (n = 4) respectively, and also, between ratNB and ratNB(-Mgat2), 40% ± 8% (n = 6) and 62% ±8% (n = 3), respectively. The n value denotes number of bands from three separate experiments for both human and rat. Horizontal lines adjacent to blots and gels indicate molecular weight standards in kDa: 250, 150, 100, 75, 50, 37, and 25, from top to bottom. Top marker was unobserved for western blot in panel C. Black and grey arrows point to MMP-2 and β-tubulin, respectively.

Candidate proteins involved in regulating cell invasiveness and proliferation were studied by gelatinase assays and western blots. Gelatin zymography of conditioned media from HuNB and HuNB(-*MGAT2)* cell lines (Figure 8A, left panel), as well as NB_1 and NB__1(-MGAT2) cell lines (Figure 8B, left panel), detected an intense band of gelatinase activity at about 65 kDa, along with some activity at higher molecular weights. In both cases, the intensity of the band was lighter for the glycosylation mutant NB cell lines than the parental NB cell lines. Western blotting using an anti-MMP2 antibody detected an immunoband at about 65 kDa in conditioned media from HuNB and HuNB(-*MGAT2)* cell lines (Figure 8A, middle panel), as well as NB_1 and NB__1(-*MGAT2*) cell lines (Figure 8B middle panel), indicating that the intense band observed from the gelatin gels was the MMP-2 protein. MMP-2 was not detected in whole cell lysates by Western blots. In addition, whole cell lysates were obtained from cells generating the conditioned medium. In both cases, coomassie blue-stained gels confirmed that similar amounts of protein were loaded for the human (Figure 8A, C, right panels) and rat (Figure 8B, D, right panels) NB cell lines. The percent of reduction in MMP-2 activity between the glycosylation mutant and parental cell lines was 29% ± 4% (n = 7) and 40% ± 8% (n = 6), while the percent of reduction in MMP-2 protein levels was 37% ± 11% (n = 4) and 62% ±8% (n = 3) for human and rat, respectively. Therefore, these results support that the decrease in MMP-2 protein levels contribute to reduced MMP-2 activity.

### 3.9. EGFR and Gab-2 Protein Levels in Parental and Mutant Cell Lines

**Figure 9:**
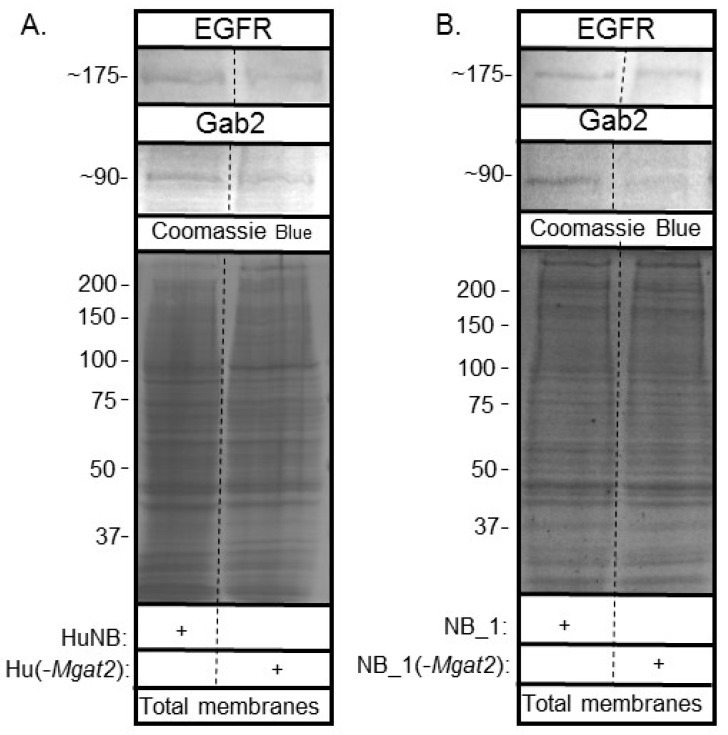
Lowered complex type N-glycans by NB cells altered EGFR and Gab2 protein levels. Western blots of EGFR (top panels) and Gab2 (middle panels) of total membranes from parental and mutant human (**A**) and rat (**B**) cell lines, as indicated. Coomassie blue stained gels (bottom panels) show that equal amounts of protein were loaded. Relative molecular weights (in KDa) of EGFR and Gab2 are next to western blots. Molecular weights (in KDa) of standards are adjacent to coomassie blue stained gel. Experiments were performed three times.

Since EGFR has been shown to be overexpressed in the HuNB cell line and NB tissues [17,18], total membranes were obtained from the various NB cell lines to determine whether EGFR and Gab2 had varied protein levels. EGFR is a receptor protein which signals the rate of cell proliferation and invasiveness in cells [17,18]. Gab2, a scaffolding protein, can amplify EGFR signaling [20,21]. Western blots of EGFR and Gab2 of human (Figure 9A) and rat (Figure 9B) NB cell lines showed immunobands of slightly greater intensity in parental NB cell lines than their corresponding glycosylation mutant cell lines. In both cases, the amount of protein loaded were similar, based on the coomassie blue-stained gels. Thus, increased hybrid and oligomannose types of N-glycans slightly reduce EGFR and Gab-2 protein levels. 

## 4. Discussion

Engineered neuroblastoma (NB) cell models, in which the N-glycosylation pathway is altered, provide a promising approach to identify roles of N-glycan types in development and progression of NB. To date, the CRISPR/Cas9 technique was employed to silence the *MGAT2* gene in human and *Mgat2* gene in rat [13,25] NB cell lines. Lectin binding studies, and also, western blots of Kv3.1b, a GnT-II (*MGAT2*) substrate [32,35], verified that both human and rat NB cell lines predominantly expressed hybrid type N-glycans, along with some oligomannose type N-glycans, and were void of complex type N-glycans. Further, the hybrid N-glycan attached to Kv3.1b expressed in the HuNB(-*MGAT2*) cell line, as well as the rat NB_1(-*Mgat2*) cell line [25], had the α1–3 mannose removed (or less than 4 mannose residues) [38] since it was insensitive to Endo H. Additionally, changes in the N-glycosylation pathway due to silencing *MGAT2* could be partially recovered by transient transfection with mouse *MGAT2,* as transfection efficiency is low in NB cells. It was also discovered that MMP-2 activity and protein levels were markedly reduced when complex-type N-glycans were lowered, indicating that MMP-2 protein expression was a contributor to the reduction of MMP-2 activity. Both human and rat NB cell lines had similar trends in that they slowed cell proliferation and cell invasiveness as complex type N-glycans were lowered. Therefore, these two engineered glycosylation mutant cell models which lack complex type N-glycans, along with their parental cell lines, will greatly assist in identifying the relevance of GnT-II (*MGAT2*) protein substrate in altering NB cellular properties.

Previously, we showed that less processed N-glycans, such as hybrid and oligomannose types of N-glycans, suppressed cell proliferation and cell invasiveness in rat B35 NB (NB_1) cells [13]. Since NB is biologically and clinically heterogeneous, we undertook a similar study using human Be(2)C NB (HuNB) cells. In both cases, it was observed that cells with decreased levels of complex type N-glycans (HuNB(-*MGAT2*) and RatNB_1(-*Mgat2*)), compared to those with increased levels of complex type N-glycans (HuNB and RatNB_1), diminished cell invasiveness and enhanced cell–cell adhesion to similar degrees, while cell proliferation was somewhat different. Nonetheless, the trends were similar, such that increases in less processed N-glycans suppressed NB cellular properties. It should also be mentioned that hybrid type N-glycans were more prevalent, while several β1,6-branched N-glycan structures, complex type N-glycans, were less prevalent in cells (SY5Y) acquired from a low-risk than cells (NLF) from a high-risk NB [12]. Our current study shares the importance of increased hybrid type in suppression of the malignant phenotype, as substitution of complex with hybrid and oligomannose types of N-glycans could lessen the aberrant cellular properties. 

Past studies reported the overexpression of EGFR in the HuNB cell line, as well as the presence of EGFR in NB tissues [17,18]. Since cell growth assays revealed that both human and rat [13] NB cells expressing lower levels of complex type N-glycans grew at slower rates than those cells with more complex type N-glycans, EGFR expression was measured in the parental and glycosylation mutant cell lines. Interestingly, the levels of EGFR were slightly reduced in the glycosylation mutant cell lines relative to their respective parental cell line. Further, Gab-2, an amplifier of EGFR signaling [20,21], was more highly expressed in the parental NB cells than the glycosylation mutant NB cells (Figure 9). Taken together, changes in the levels of EGFR and Gab-2 appear to accompany modifications in cell growth and proliferation in NB cell lines due to altering the ratio of complex-type to hybrid and oligomannose types of N-glycans. Further, these studies encourage a more thorough analysis of the EGFR signaling pathway.

EGFR is a GnT-II protein substrate [39,40,41,42]. The N-glycans of EGFR are involved in ligand binding, dimerization, and tumor promotion [39,40,41,42]. It was also shown that enhanced branching of the N-glycans of EGFR modulated cell growth and proliferation [43]. Since NB cells predominantly expressing hybrid type N-glycans grew at a slower rate compared to their counterparts expressing predominantly complex type N-glycans, it seems reasonable to suspect that EGFR signaling is diminished when complex type is replaced with hybrid and oligomannose types of N-glycans in NB. Further reduction in EGFR signaling is supported by a recent study that revealed inhibition of N-glycosylation by tunicamycin in head and neck cancer cells inhibited tumorigenesis by triggering endoplasmic reticulum (ER) stress [44]. As such, it may be that inhibiting conversion of hybrid to complex types of N-glycans results in accumulation of glycosylated protein in lumen of ER, resulting in ER stress and thereby suppression of tumorgenicity.

The matrix metalloproteinases (MMPs) are involved in remodeling of the ECM, and also, they are influential in cancer progression by enhancing cell growth and cell invasion [45]. Based on zymography and western blotting, MMP-2 was observed in both human and rat NB cell lines, and also, the decrease in MMP-2 protein activity could be explained by decreased MMP-2 protein levels. The expression of MMP-2 in the NB cell lines is supported by past studies that show that MMP-2 has been found in all tumors examined [46]. It has also been established that the lack of MMP-2 in mice reduces tumor growth [47]. Of interest, various anti-cancer compounds have been shown to cause ER stress and lower MMP levels [48,49], it is possible that the lack of MGAT1 expression leads to ER stress as glycosylated proteins build up in the lumen of ER. Poly-N-acetyllactosamines (PLAs) on N-glycans induce an invasive phenotype [50]. Further, it was reported that knockdown of β-1, 3-N-acetyl glucosaminyltranferase-8 in gastric cancer cells could suppress invasive potential, which was correlated with reduced MMP-2 expression and activity levels [51]. Future studies are required to determine whether decreased MMP-2 protein levels are solely responsible for the lowered MMP-2 activity, the effect of ER stress on MMP-2 levels, and whether the changes in MMP-2 are due to a given set of PLA on complex-type N-glycans. 

## 5. Conclusion

In conclusion, the current study showed that cell proliferation and cell invasiveness were decreased while cell–cell adhesion was strengthened when the N-glycosylation pathway was altered to raise hybrid and oligomannose types and lower complex types of N-glycans in two separate NB cell lines. Further, decreased protein expression and activity of MMP-2 was correlated with declines in cell growth and cell invasiveness in both cell lines. As such, the decreased ratio of complex N-glycans to less processed N-glycans, such as hybrid and oligomannose types, suppressed cell proliferation and invasiveness in NB by altering ECM remodeling. These studies support glycans as potential avenues for anti-cancer therapy (or suppressing NB).
